# The joint Nagasaki–Würzburg approach to challenges and perspectives in neuropsychiatric and regenerative research

**DOI:** 10.1007/s00702-020-02263-2

**Published:** 2020-10-10

**Authors:** Jürgen Deckert, Hiroki Ozawa

**Affiliations:** Department of Psychiatry, Julius-Maximilians-University Würzburg and Nagasaki University, Nagasaki, Japan

The Nagasaki and Würzburg exchange and cooperation in the field of medicine goes back to the Würzburg physician and botanist Philip Franz von Siebold who lived in Nagasaki from 1823 to 1830 and again from 1859 to 1862. He was born into a famous Würzburg physicians’ family which provided Würzburg Medical Faculty with chairs in surgery and gynecology. Philip Franz von Siebold joined the Dutch East-Indian company in 1822 and arrived on the island of Dejima in the bay of Nagasaki on August 11, 1823. The Dutch settlement on Dejima was the only foreign settlement permitted in Japan until the mid nineteenth century. During his first stay, Philip Franz von Siebold was allowed to treat Japanese patients and to communicate with Japanese physicians such as Keisuke Ito, Mizutani Sugeroku, Ōkochi Zonshin and Katsuragawa Hoken on the Nagasaki mainland. He is thus considered by Nagasaki Medical Faculty as one of its founders together with the Dutch physician Johannes Lijdius Catharinus Pompe van Meerdervoort who lived in Nagasaki from 1857 to 1862 and established the first Western-style hospital and a formal medical school in 1861. Kusumoto-Ine, the daughter of Philip Franz von Siebold with his Japanese wife Sonogi O-Taki, attended this medical school and became a well-known Japanese physician while his German son Alexander Gustav von Siebold lived in Japan from 1870 to 1911 and became a cofounder of the Japanese Red Cross.

Based on this history Nagasaki and Würzburg University started a formal student exchange program in 1996, signed by the President T. Berchem, the Dean K. Wilms, the President T. Yokoyama and the Dean S. Nagataki. Since then the academic exchange has been promoted particularly by Prof. C. Reiners, Prof. M. Tomonaga, Prof. S. Yamashita and the president Prof S. Kono. In 2006, a formal academic cooperation agreement has been signed by the President A. Haase and the President H. Saito. Over the years and partly funded by DFG and JSPS a number of bilateral research projects with reciprocal research stays in particular between the two psychiatric departments (H. Ozawa, M. Bartl and S. Ono) and recently the two anatomy institutes (I. Takashi) have been successfully performed (Ozawa et al. 1993, 1999; Bartl et al. 2013; Morimoto et al. 2018; Takashi et al. 2020). In addition to scientific contacts numerous personal contacts have developed including marriages as in Siebold’s time. To establish a formal strategic partnership at the educational, research and clinical level since 2016 regular joint symposia have taken place, the most recent and 3rd one on April 15 and 16, 2019 at Nagasaki Medical School. This 3rd joint symposium focused on “Advances in Comprehensive cancer therapy” with sessions on immunotherapy and novel drug targets, mechanisms of radiation-induced cancer and improving the effectiveness of radionuclide therapy, contribution of imaging and radionuclide therapy to precision oncology, stem cells—use in comprehensive cancer therapy and improving palliative care.

Results of research presented at this symposium relevant to the lines of research followed in neuropsychiatry by the two psychiatric departments are presented in this special issue and complemented by a number of partly joint research results and reviews on challenges in neuropsychiatric and regenerative research. The manuscripts are thus providing an agenda for future cooperation between the two universities in the context of the recently (2019) established formal bilateral postdoc externship program funded by the two medical faculties. Both medical faculties and in particular the two psychiatric departments strongly believe that to overcome the challenges in a global world as highlighted by the recent SARS-CoV-2 pandemia, strategic partnerships including the educational, research and clinical level are essential.

One major challenge during the current SARS-CoV-2 pandemia for all people and in particular for people with neuropsychiatric disorders is social isolation. Modern digital media have been employed to compensate for this at least to a certain degree and it is expected that they will change psychiatry for good (Gründahl et al. 2020). However, they will not be able to replace personal contacts across nations and cultures completely.

We therefore hope that the joint Nagasaki–Würzburg approach initiated by visionaries like Philip Franz von Siebold and his Japanese colleagues 200 years ago may serve as a blueprint for numerous other bi- and multilateral approaches to global challenges in the face of isolationistic and nationalistic approaches during the current SARS-CoV-2 pandemia.
